# EHMTI-0380. The association of migraine susceptibility loci with severe migraine characteristics in a clinic-based migraine sample

**DOI:** 10.1186/1129-2377-15-S1-H1

**Published:** 2014-09-18

**Authors:** A Esserlind, AF Christensen, S Steinberg, N Grarup, O Pedersen, T Hansen, T Werge, T Folkmann-Hansen, LL Husemoen, A Linneberg, E Budtz-Jorgensen, M Lurenda Westergaard, H Stefansson, J Olesen

**Affiliations:** 1 Neurology, Danish Headache Center, Glostrup, Denmark; 2 deCODE, deCODE Genetics, Reykjavik, Iceland; 3 Faculty of Health and Medical Sciences Copenhagen University, Novo Nordisk Foundation Center for Basic Metabolic Research, Copenhagen, Denmark; 4 Clinical Medicine University of Copenhagen, Institute of Biological Psychiatry MHC Sct.Hans, Copenhagen, Denmark; 5 Capital Region of Denmark, Research Centre for Prevention and Health, Copenhagen, Denmark; 6 Glostrup University Hospital, Department of Clinical Experimental Research, Copenhagen, Denmark; 7 University of Copenhagen, Faculty of Health and Medical Sciences, Copenhagen, Denmark

## Introduction and aim

Migraine with typical aura (MTA) and migraine without aura (MO) are common neurological disorders with complex inheritance. Recent efforts have identified 12 independent loci at which single nucleotide polymorphisms (SNPs) have shown to confer risk of migraine (Antilla V. Nat.Genet 2013). The objective of this study was to investigate whether these SNPs could be replicated in a Danish clinic based migraine sample and to test if the risk-alleles are associated with severe migraine traits.

## Methods

Semi-structured migraine interviews based on a validated questionnaire, blood samples and genotyping were performed on 1806 unrelated migraineurs from the Danish Headache Center, Glostrup Hospital. The control group consisted of 6415 individuals with no history of migraine. Association analyses were carried out using logistic regression. The primary endpoints were regarded as a proxy for severe migraine traits (early onset of migraine; many lifetime attacks; prolonged migraine attacks and chronification of migraine) and tested against the 12 SNPs and a combined genetic risk score.

## Results

Five out the 12 previously reported loci were replicated in our sample. The association was significant only for those with migraine without aura. Following correction for statistical testing, five SNPs showed nominal association with the severe migraine traits:‘early onset of migraine’,‘prolonged migraine attacks’ and ‘many lifetime attacks’.

## Conclusion

Our study confirms previous findings on the association of several SNPs with migraine. The association results with severe features, albeit nominal, suggests that the previously reported migraine risk alleles may be implicated in the development of severe migraine characteristics.

No conflict of interest.

